# Projected long-term effects of colorectal cancer screening disruptions following the COVID-19 pandemic

**DOI:** 10.7554/eLife.85264

**Published:** 2023-05-02

**Authors:** Pedro Nascimento de Lima, Rosita van den Puttelaar, Anne I Hahn, Matthias Harlass, Nicholson Collier, Jonathan Ozik, Ann G Zauber, Iris Lansdorp-Vogelaar, Carolyn M Rutter

**Affiliations:** 1 RAND Corporation Santa Monica United States; 2 Erasmus MC Rotterdam Netherlands; 3 Memorial Sloan Kettering Cancer Center New York United States; 4 Argonne National Laboratory Lemont United States; 5 Fred Hutchinson Cancer Center Seattle, WA United States; McGill University Canada; McGill University Canada

**Keywords:** colorectal cancer, COVID-19, microsimulation, cancer screening, comparative modeling, Human

## Abstract

The aftermath of the initial phase of the COVID-19 pandemic may contribute to the widening of disparities in colorectal cancer (CRC) outcomes due to differential disruptions to CRC screening. This comparative microsimulation analysis uses two CISNET CRC models to simulate the impact of ongoing screening disruptions induced by the COVID-19 pandemic on long-term CRC outcomes. We evaluate three channels through which screening was disrupted: delays in screening, regimen switching, and screening discontinuation. The impact of these disruptions on long-term CRC outcomes was measured by the number of life-years lost due to CRC screening disruptions compared to a scenario without any disruptions. While short-term delays in screening of 3–18 months are predicted to result in minor life-years loss, discontinuing screening could result in much more significant reductions in the expected benefits of screening. These results demonstrate that unequal recovery of screening following the pandemic can widen disparities in CRC outcomes and emphasize the importance of ensuring equitable recovery to screening following the pandemic.

## Introduction

The novel SARS-Cov-2 (COVID-19) pandemic has resulted in major health consequences across the globe. In addition to the over 1 million COVID-19 deaths in the United States (Johns Hopkins Univerity & Medicine Coronavirus Resource Center, 2022), the pandemic has also contributed to steep declines in cancer screening, most notably in the early phases of the pandemic due to government-mandated shutdowns of non-emergency medical services (Gupta et al., 2020). It is estimated that colorectal cancer (CRC) screening decreased by 85% in the United States during the early phase of the pandemic, from March through April 2020 (London et al., 2022). The pandemic continues to affect CRC screening and diagnosis through staff shortages that reduce capacity at gastroenterology clinics and patient hesitancy to seek care (Wilensky, 2022; Del Vecchio Blanco et al., 2020). Despite cancer screening reopening efforts, CRC screening has not yet returned to pre-pandemic levels (Ong, 2021).

CRC remains the second-leading cause of cancer deaths in the United States, with approximately 153,020 new cases and 52,550 deaths estimated in the year 2023 (Siegel et al., 2023). There is clear evidence that screening has a major impact on reducing the burden of CRC (Edwards et al., 2010; Zauber et al., 2012) and that it is cost-effective (Knudsen et al., 2021; Lansdorp-Vogelaar et al., 2011). The current United States Preventive Task Force (USPSTF) report recommends multiple screening options, including annual fecal immunochemical tests (FIT) and colonoscopy every 10 years for average-risk individuals (Davidson et al., 2021). However, CRC screening uptake was of concern even before the pandemic, with CRC screening rates well below the goal of 70.5% for Healthy People 2020 and the National Colorectal Cancer Roundtable goal of 80% by 2018 (Shapiro et al., 2021). Low rates of CRC screening have been exacerbated by the COVID-19 pandemic, and delays in screening will result in delays in diagnosis, stage progression, and increased CRC mortality.

The pandemic may also further exacerbate existing disparities related to screening. The burden of unemployment and associated loss of access to healthcare varies across different racial and ethnic groups (Marcondes et al., 2021). Because of this, the pandemic may contribute to widening disparities in cancer outcomes. A recent analysis using National Health Interview Survey (NHIS) data postulated that unemployment was adversely associated with being up-to-date with screening, with only 16.7% of unemployed individuals participating in recent CRC screenings, only 48.5% of whom were up-to-date with CRC screening (Fedewa et al., 2022).

The objective of this study is to estimate the impact of ongoing screening and treatment disruptions induced by the COVID-19 pandemic on long-term CRC outcomes. We examine 25 scenarios that reflect different levels of pre-pandemic adherence to colonoscopy and FIT screening to assess how unequal recovery in screening may contribute to widening disparities in CRC lifetime outcomes.

## Methods

This paper uses two independently developed microsimulation models of CRC, CRC-SPIN and MISCAN-Colon, to estimate the effects of pandemic-induced disruptions in colonoscopy screening for eight pre-pandemic average-CRC risk population cohorts in the United States. CRC-SPIN and MISCAN-Colon models are part of the National Cancer Institute’s CISNET consortium and describe the natural history of CRC in an unscreened population based on the adenoma-carcinoma sequence. Detailed descriptions of these models and underlying assumptions may be found elsewhere (Knudsen et al., 2021; Loeve et al., 1999; Rutter et al., 2019, van Hees et al., 2014). We consider variations on two commonly used screening strategies in the USPSTF recommendations during the onset of the pandemic in March 2020 (Knudsen et al., 2016): Decennial colonoscopy from age 50 to 70 and annual FIT from age 50 to 75, with diagnostic colonoscopy after a positive FIT.

### Cohorts

We simulated eight pre-pandemic population cohorts that represent average-risk individuals in the United States, defined by both cohort members’ age in April 2020 and their pre-pandemic screening regimens: (i) unscreened 50-year-olds (U50), (ii) unscreened 60-year-olds (U60), (iii) colonoscopy screening-adherent 60-year-olds (C60, who received their first screening colonoscopy at age 50 but have not yet had a colonoscopy at age 60), (iv) FIT screening-adherent 60-year-olds (F60, who performed annual FIT from age 50 to 59), (v) FIT screening semi-adherent 60-year-olds (f60) – those who received biannual FIT from age 50 to 56, (vi) unscreened 70-year-olds (U70), (vii) colonoscopy screening-adherent 70-year-olds (C70, who received screening colonoscopies at age 50 and 60), and (viii) FIT screening-adherent 70-year-olds (F70, who performed annual FIT from age 50 to 69). We simulated 10 million individuals within each cohort to reduce the stochastic variability in our runs and to ensure sufficient precision in our estimates. For each cohort, we simulated three sets of post-pandemic scenarios: no disruption, delays, and no screening.

### Screening regimens under no disruption

The *no-disruption* scenarios simulate post-pandemic screening scenarios for each cohort in the counterfactual scenario where no pandemic-induced screening disruptions occurred. In no-disruption scenarios, all these cohorts would have been screened during the pandemic first lockdowns in March 2020. Cohorts with colonoscopy and FIT adherent individuals (U50, C60, F60 C70, F70) continue to follow guideline-recommended strategies strictly, with no delays. Cohorts with delayed initiation (U60, U70) begin screening late but otherwise follow guideline-recommended strategies with no delays but without any additional screening beyond the usual stopping age. Finally, for the *FIT-semi-adherent 60-year-olds* (f60), we simulate resumption of biannual FIT at age 60, continuing to age 75.

### Pandemic-induced disruptions in CRC screening

#### Delays

The pandemic has been shown to affect CRC outcomes through *delays* in screening. Screening colonoscopy and FIT are assumed to be delayed for a set duration of months starting at the onset of the COVID-19 pandemic in April 2020. Short-term screening delays may have occurred for a series of reasons. First, elective procedures were postponed during the first months of the pandemic. The cancellation of elective procedures caused a sharp decline in CRC screening exams during the initial phase of the pandemic (Gupta and Lieberman, 2020). To represent the full spectrum of delays caused by the pandemic – either due to cancellation of elective procedures or disruption in access to healthcare – we consider three sets of delays: a 3-, 9-, or 18-month delay in screening, which we label as *short-term* delays. For each delay scenario, the delay was applied on the first post-March 2020 screening exam and carried forward to any subsequent exams.

Second, the pandemic may have caused *long-term delays* in CRC screening. While the recovery in screening rates among insured individuals was rapid, (Choy et al., 2022) the pandemic also caused a sharp economic recession. The uneven recovery in labor force participation has the potential to cause disparities in access to healthcare in the United States due to unemployment and discontinuation of health insurance. To examine these longer-term effects of the pandemic, we consider scenarios where screening is paused for an extended period. For the 50- and 60-year-old cohorts, we simulated scenarios where screening is discontinued until the start age of Medicare enrollment (65 years). For 70-year-olds, we consider a scenario where screening is only resumed at age 75 – 5 years after the pandemic onset.

#### Screening regimen switching

The pandemic may also affect CRC behavior via screening *regimen switching –* that is, changing from a colonoscopy screening regimen to one based on FIT. There is evidence that during the pandemic some patients switched from colonoscopy to FIT (Fedewa et al., 2022) to reduce the need for in-person endoscopy procedures. Considering this possibility, we model scenarios where individuals who initially participated in a regimen of screening colonoscopy (C60 and C70) permanently switch from decennial colonoscopy to annual FIT screening as a boundary case. While one might expect pandemic-induced regimen switching to be temporary, permanent switching can serve as a boundary case for our analysis – that is, the effect of short-term regimen switching is expected to be lower than the effect of permanent regimen switching.

#### Screening discontinuation

We also simulate scenarios where screening is completely discontinued after the pandemic onset as the most consequential boundary case scenario. While only a small (unknown) proportion of individuals will discontinue screening after the pandemic, this scenario serves as an upper bound for the worst possible disruption in CRC screening following the pandemic.

#### Scenarios

Each of the scenarios simulated in this study results from the combination of a pre-pandemic population cohort, a no-disruption screening scenario that serves as a counterfactual, and one or more screening disruptions (i.e. switching to FIT screening occurred in tandem with short-term delays). Table 1 lists those combinations and the scenario labels used in this analysis. We code our scenarios as *[pre-pandemic screening cohort] | [post-pandemic disruptions]*.

**Table 1.** Study cohorts and scenarios.

Cohort	No-disruption counterfactual	CRC screening disruption scenario	
		Description	Label
Unscreened 50-year-olds (U50)	Decennial COL from age 50 to 70	Short-term delays of [d] months^*^	U50 | C[d]m
		Long-term delay (COL at age 65 and 75)	U50 | C@65
	Annual FIT from age 50 to 75	Short-term delays^*^	U50 | F[d]m
Unscreened 60-year-olds (U60)	Decennial COL from age 60 to 70	Short-term delays^*^	U60 | C[d]m
		Long-term delay (COL at age 65 and 75)	U60 | C@65
	Annual FIT from age 60 to 75	Short-term delays^*^	U60 | F[d]m
COL-adherent 60-year-olds (C60)	Decennial COL from age 50 to 70	Short-term delays^*^	C60 | C[d]m
		Switch to annual FIT and short-term delays	C60 | F[d]m
		Long-term delay (COL at age 65 and 75)	C60 | C@65
		Discontinue screening	C60 | U
FIT-adherent 60-year-olds (F60)	Annual FIT from age 50 to 75	Short-term delays^*^	F60 | F[d]m
		Discontinue screening	F60 | U
FIT-semi-adherent 60-year-olds (f60)	Biannual FIT from age 50 to 56, annual FIT from age 60 to 75	Short-term delays^*^	f60 | F[d]m
		Discontinue screening	f60 | U
Unscreened 70-year-olds (U70)	COL at age 70	Short-term delays^*^	U70 | C[d]m
		Long-term delay (COL at age 75)	U70 | C@75
	Annual FIT from age 70 to 75	Short-term delays	U70 | F[d]m
COL-adherent 70-year-olds (C70)	Decennial COL from age 50 to 70	Short-term delays^*^	C70 | C[d]m
		Switch to annual FIT and short-term delays	C70 | F[d]m
		Long-term delay Perform COL at age 75	C70 | C@75
		Discontinue screening	C70 | U
FIT-adherent 70-year-olds (F70)	Annual FIT from age 50 to 75	Short-term delays^*^	F70 | F[d]m
		Discontinue screening	F70 | U

Notes: This table presents the scenarios considered in this study. Each scenario corresponds to a combination of a population cohort, indicated by their age during the first COVID-19 lockdowns (March 2020), a pre-pandemic, and a post-pandemic screening regimen. The scenarios aim to represent possible combinations of screening regimens followed in the United States. The first letter in the scenario code represents screening before the pandemic and the second letter represents screening after the pandemic.

* Delays of 3, 9, and 18 months. Letter d stands for the number of months of delays.

#### Outcomes

The primary measure used to assess the benefit of CRC screening programs is the expected lifetime life-years gained (LYG) from screening. All outcomes in this study correspond to expected value of life-years (LY) across the US population with average CRC risk. This study investigates the extent to which benefits from screening are expected to be lost due to pandemic-induced disruptions to CRC screening. Therefore, we calculated the total number of LY for each cohort and scenario, including the number of LY under no screening (LYNS) and the number of LY under no disruptions (LYND). LYNS is computed by simulating the cohort in the absence of CRC screening and LYND is computed by simulating the same cohort under an ideal screening scenario where no disruptions to screening happened, as defined in Table 1.

The key outcome estimated in this study is the expected number of LY lost (LYL) due to disruptions in screening, defined as LYL= LYND−LY. The hypothetical number of LY gained (LYG) from screening under no disruptions are $LYG_{no\;disruption}\;=\;LYND-LYNS$. Finally, we compute the percentage of life-years gained or lost due to disruption as $\%LYLost=100*LYL/LYG_{nodisruption}$ . The first outcome measure (*LYL*) is an absolute measure of the loss of screening benefit due to pandemic disruptions. The percent LY lost due to disruptions indicates the share of screening benefit lost due to the pandemic. Following the previous analyses, we present all outcomes as LY per 1000 individuals or life days per person. We compute each of those outcomes separately for each model and report the range of outcomes observed across both models. In addition to LY outcomes, we present lifetime number of CRC cases over the remaining lifetime of individuals and number of CRC deaths (Supplementary files 2 and 3).

### Test characteristics

Table 2 specifies sensitivity and specificity assumptions underlying colonoscopy and FIT exams evaluated in this study. Our main results present colonoscopy sensitivity following assumptions used in the analysis that informed the most recent USPSTF screening recommendations (Zauber et al., 2008). In addition, we simulate all screening disruption scenarios under assuming lower colonoscopy sensitivity.

**Table 2.** Per lesion test sensitivity and specificity.

	Sensitivity*				Specificity†
Test	Adenoma1–5 mm	Adenoma6–9 mm	Adenoma ≥10 mm	Preclinical cancer	
Colonoscopy, high sensitivity‡	0.75	0.85	0.95	0.95	0.86
Colonoscopy, low sensitivity§	0.55	0.70	0.90	0.95	0.86
FIT¶					
MISCAN	0.00	0.114	0.159	0.62565/0.886	0.97
CRC-SPIN	0.05	0.15	0.22	*0.74	0.97

Notes: This table presents the assumed test characteristics. We simulated two colonoscopy sensitivity scenarios seeking to represent a range of colonoscopy sensitivity of gastroenterologists in the United States.

* Sensitivity is for lesions within reach of the scope. We assume the same test characteristics for follow-up and surveillance colonoscopy as for screening colonoscopy.

† For FIT, the lack of specificity reflects detection of bleeding from other causes. We assume other-cause bleeding is independent of adenoma status. For colonoscopy, the lack of specificity reflects detection of non-adenomatous lesions, but specificity is handled in post-processing in cost-effectiveness analyses. Since this study does not consider burden outcomes, specificity is not considered in this paper. Specificity values were obtained from Lin et al., 2021.

‡ Baseline scenarios used in Zauber et al., 2008.

§ In line with low-sensitivity scenarios compatible with Rutter et al., 2022.

¶ CRC-SPIN uses per-person test sensitivity for stool-based tests that are based on the size of the most advanced lesion. To account for the likelihood that a person with multiple adenomas is more likely than a person with only one to have a positive stool test, MISCAN uses lesion-based sensitivities instead of person-based sensitivities. Lesion-based sensitivities were derived by calibrating the person-based sensitivities to the number of people having one or more small/medium/large adenomas or cancers detected by stool-based testing with diagnostic colonoscopy, divided by those having one or more small/medium/large adenomas or cancers detected by colonoscopy screening.

### CRC surveillance

We assume that individuals with an adenoma detected undergo colonoscopic surveillance according to the Multi-Society Task Force (MSTF) guidelines. These guidelines provide intervals for surveillance based on baseline findings and findings at the first surveillance colonoscopy. We assume that the intervals provided can be more generally expressed as the intervals based on the most recent colonoscopy (‘first-most recent colonoscopy’) and the colonoscopy prior to that (‘second-most recent colonoscopy’). In situations where the MSTF provided a range rather than a single interval, we assumed that the shortest interval would be used in routine practice. The resulting intervals are shown in Table 3.

**Table 3.** CRC surveillance intervals.

Finding at second-most recent colonoscopy^*^^†^	Finding at first-most recent colonoscopy^*^^†^	Interval^‡^ to next colonoscopy, years
No prior colonoscopy	Normal colonoscopy	See note below§
	1–2 adenomas <10 mm	7
	3–4 adenomas <10 mm	3
	10 adenomas <10 mm or any adenoma ≥10 mm	3
	>10 adenomas	1
Normal colonoscopy	Normal colonoscopy	10
	1–2 adenomas <10 mm	7
	3–4 adenomas <10 mm	3
	5–10 adenomas <10 mm or any adenoma ≥10 mm	3
	>10 adenomas	1
1–2 adenomas <10 mm	Normal colonoscopy	10
	1–2 adenomas <10 mm	7
	3–4 adenomas <10 mm	3
	5–10 adenomas <10 mm or any adenoma ≥10 mm	3
	>10 adenomas	1
3–4 adenomas <10 mm	Normal colonoscopy	10
	1–2 adenomas <10 mm	7
	3–4 adenomas <10 mm	3
	5–10 adenomas <10 mm or any adenoma ≥10 mm	3
	>10 adenomas	1
5–10 adenomas <10 mm	Normal colonoscopy	5
or	1–2 adenomas <10 mm	5
any adenoma ≥10 mm	3–4 adenomas <10 mm	3
	5–10 adenomas <10 mm or any adenoma ≥10 mm	3
	>10 adenomas	1
>10 adenomas of any size	Normal colonoscopy	5
	1–2 adenomas <10 mm	5
	3–4 adenomas <10 mm	3
	5–10 adenomas <10 mm or any adenoma ≥10 mm	3
	>10 adenomas	1

* A normal colonoscopy is one in which no adenomas, SSPs (not currently simulated), or CRC is detected.

† This table omits the case where CRC is detected at a screening, diagnostic, or surveillance colonoscopy because the CISNET CRC models do not simulate detailed events following CRC diagnosis.

‡ The Multi-Society Task Force provides a range for some intervals (e.g. the interval for 3–4 adenomas <10 mm is 3–5 years). In such cases, we selected the shortest intervals provided.

§ A person whose first screening or diagnostic colonoscopy is normal does not enter surveillance but instead resumes screening with the original modality 10 years after the normal colonoscopy. The exception to the 10-year waiting period is when the first colonoscopy is a screening colonoscopy with an *x*-year interval, where *x*>10. In that case, the next colonoscopy is in *x* years.

We assume that persons in whom adenoma(s) have been detected remain on surveillance until age 85, provided that no adenomas are detected at the last surveillance colonoscopy. If adenomas are detected, then surveillance continues according to the clinical findings at the last colonoscopy until the person has a colonoscopy with no adenomas detected. For example, if a person has a surveillance colonoscopy at age 83 and no adenomas are detected at this exam or the exam before this one, they would be recommended to have their next surveillance at age 93. Age 93 is after the surveillance stopping age of 85 and the exam prior to age 85 was negative, so they will not have any more surveillance colonoscopies after age 83. However, if the exam at age 83 instead detected 1–2 small adenomas, they would come back for their surveillance colonoscopy at age 90, because adenomas were detected at the exam at age 83.

## Results

**Figure 1.**
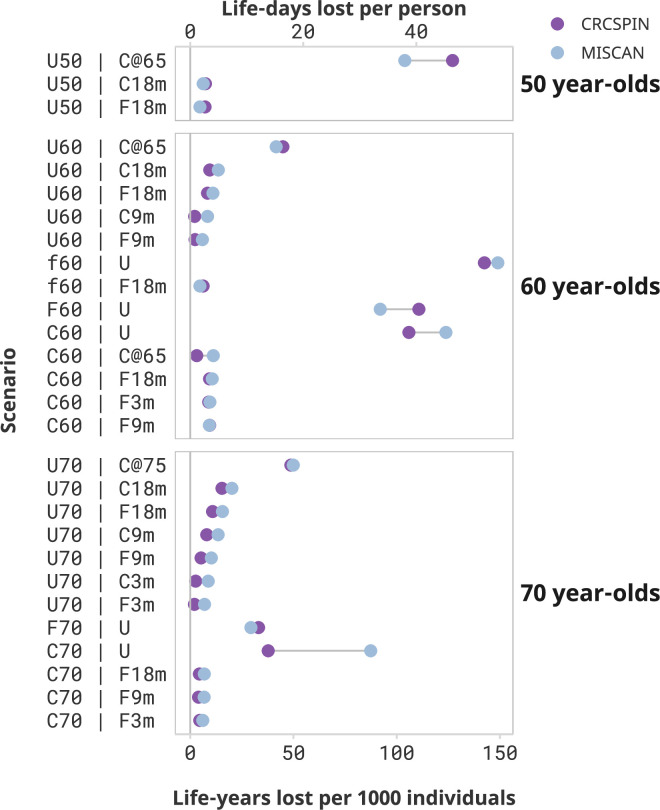
Screening benefits lost due to disruptions by cohort and scenario. Notes*:* Each dot represents the estimated life-years lost per 1000 individuals or life-days lost from one model under the high sensitivity scenario. Results are ordered from highest to lowest reduction in benefit induced by the pandemic. Scenarios that result in less than 2 life-days lost per person are omitted from this figure and presented in a Supplementary figure. This figure does not present a counterfactual no-screening scenario for the 50-year-olds.

Loss of life due to screening disruptions was the largest for cohorts with severe disruptions after the pandemic (Figure 1). Aside from not receiving any screening, the worst-case scenario for the 50-year-old cohort was to postpone screening until age 65 when they become Medicare eligible. This cohort (scenario U50 | C@65) is expected to lose 104–127 LY per 1000 individuals – a 38–42% loss in LYG compared to a no-disruption scenario where they start screening at age 50 (Table 4). This cohort would be 1.3–1.9 times more likely to have CRC over their lifetime (Supplementary file 2) and 1.6–2.0 times more likely to die with CRC (Supplementary file 3) compared to a cohort that started screening at age 50. Other disruption scenarios are predicted to have minor effects on this cohort. For example, 50-year-olds with colonoscopy screening delayed by 18 months (scenario U50 | C18m) are expected to experience a loss of 6–7 LY per 1000 individuals, and a 2% loss in LYG from screening compared to a no-disruption scenario.

**Figure 1—figure supplement 1.**
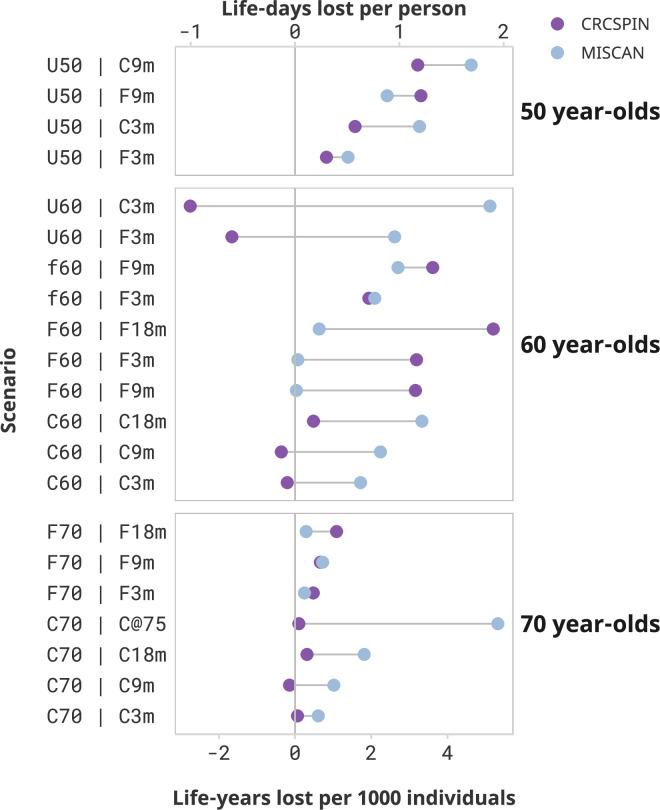
Estimated loss of life in minor disruption scenarios. Notes: Each dot represents the estimated life-years lost per 1000 individuals or life-days lost from one model under the high sensitivity scenario. Results are ordered from highest to lowest reduction in benefit induced by the pandemic. This figure supplement only shows scenarios that resulted in in less than 2 life-days lost per person. This figure does not present a counterfactual no-screening scenario for the 50-year-olds.

**Table 4.** Projected life-years (LY) per 1000 individuals.

Scenario	Model	No screening	Screening without disruptions		Screening with disruptions		Loss due to disruptions	
		LY[a]	LY[b]	LYG[b-a]	LY[c]	LYG[c-a]	LY[b-c]	% LYG loss[(b-c)/b]
U50 | C3m	CRCSPIN	31,595	31,893	299	31,892	297	2	1
	MISCAN	31,222	31,494	273	31,491	270	3	1
U50 | C9m	CRCSPIN	31,595	31,893	299	31,890	295	3	1
	MISCAN	31,222	31,494	273	31,490	268	5	2
U50 | C18m	CRCSPIN	31,595	31,893	299	31,886	291	7	2
	MISCAN	31,222	31,494	273	31,488	266	6	2
U50 | C@65	CRCSPIN	31,595	31,893	299	31,766	172	127	43
	MISCAN	31,222	31,494	273	31,390	169	104	38
U50 | F3m	CRCSPIN	31,595	31,866	271	31,865	270	1	0
	MISCAN	31,222	31,483	261	31,481	259	1	1
U50 | F9m	CRCSPIN	31,595	31,866	271	31,862	268	3	1
	MISCAN	31,222	31,483	261	31,480	258	2	1
U50 | F18m	CRCSPIN	31,595	31,866	271	31,858	264	7	3
	MISCAN	31,222	31,483	261	31,478	256	5	2
U60 | C3m	CRCSPIN	23,336	23,557	221	23,560	224	-3	-1
	MISCAN	23,114	23,309	195	23,304	190	5	3
U60 | C9m	CRCSPIN	23,336	23,557	221	23,555	219	2	1
	MISCAN	23,114	23,309	195	23,301	187	8	4
U60 | C18m	CRCSPIN	23,336	23,557	221	23,547	211	10	4
	MISCAN	23,114	23,309	195	23,296	182	14	7
U60 | C@65	CRCSPIN	23,336	23,557	221	23,512	176	45	20
	MISCAN	23,114	23,309	195	23,267	153	42	21
U60 | F3m	CRCSPIN	23,336	23,528	191	23,529	193	-2	-1
	MISCAN	23,114	23,291	177	23,288	174	3	1
U60 | F9m	CRCSPIN	23,336	23,528	191	23,525	189	2	1
	MISCAN	23,114	23,291	177	23,285	171	6	3
U60 | F18m	CRCSPIN	23,336	23,528	191	23,519	183	8	4
	MISCAN	23,114	23,291	177	23,280	166	11	6
C60 | C3m	CRCSPIN	23,243	23,541	298	23,541	299	0	0
	MISCAN	23,077	23,320	243	23,318	241	2	1
C60 | C9m	CRCSPIN	23,242	23,541	298	23,541	299	0	0
	MISCAN	23,077	23,319	243	23,317	240	2	1
C60 | C18m	CRCSPIN	23,242	23,541	298	23,540	298	0	0
	MISCAN	23,077	23,320	243	23,317	239	3	1
C60 | F3m	CRCSPIN	23,242	23,541	298	23,532	289	9	3
	MISCAN	23,077	23,320	243	23,310	233	10	4
C60 | F9m	CRCSPIN	23,243	23,541	298	23,532	289	9	3
	MISCAN	23,077	23,319	243	23,310	233	9	4
C60 | F18m	CRCSPIN	23,243	23,541	298	23,532	289	9	3
	MISCAN	23,077	23,320	243	23,309	232	11	4
C60 | C@65	CRCSPIN	23,243	23,541	298	23,538	295	3	1
	MISCAN	23,077	23,320	243	23,308	231	11	5
C60 | U	CRCSPIN	23,243	23,541	298	23,435	192	106	36
	MISCAN	23,077	23,320	243	23,195	118	125	51
F60 | F3m	CRCSPIN	23,307	23,579	272	23,576	269	3	1
	MISCAN	23,144	23,377	234	23,377	233	0	0
F60 | F9m	CRCSPIN	23,306	23,578	272	23,575	269	3	1
	MISCAN	23,143	23,376	234	23,376	234	0	0
F60 | F18m	CRCSPIN	23,307	23,578	272	23,573	267	5	2
	MISCAN	23,143	23,377	234	23,376	233	1	0
F60 | U	CRCSPIN	23,307	23,579	272	23,467	160	111	41
	MISCAN	23,143	23,376	234	23,283	141	93	40
f60 | F3m	CRCSPIN	23,314	23,562	249	23,560	247	2	1
	MISCAN	23,136	23,355	219	23,353	217	2	1
f60 | F9m	CRCSPIN	23,314	23,563	249	23,559	245	4	1
	MISCAN	23,136	23,355	219	23,352	216	3	1
f60 | F18m	CRCSPIN	23,313	23,562	249	23,556	242	6	3
	MISCAN	23,136	23,355	219	23,350	214	5	2
f60 | U	CRCSPIN	23,313	23,562	249	23,419	105	143	58
	MISCAN	23,134	23,353	219	23,203	68	150	69
U70 | C3m	CRCSPIN	15,973	16,102	128	16,099	126	3	2
	MISCAN	15,748	15,866	117	15,857	108	9	8
U70 | C9m	CRCSPIN	15,973	16,102	128	16,094	120	8	6
	MISCAN	15,748	15,866	117	15,852	103	14	12
U70 | C18m	CRCSPIN	15,973	16,102	128	16,086	113	16	12
	MISCAN	15,748	15,866	117	15,845	97	21	18
U70 | C@75	CRCSPIN	15,973	16,102	128	16,052	79	50	39
	MISCAN	15,748	15,866	117	15,815	66	51	43
U70 | F3m	CRCSPIN	15,973	16,069	95	16,067	93	2	2
	MISCAN	15,748	15,840	92	15,833	85	7	8
U70 | F9m	CRCSPIN	15,973	16,069	95	16,063	90	5	6
	MISCAN	15,748	15,840	92	15,830	81	10	11
U70 | F18m	CRCSPIN	15,973	16,069	95	16,058	84	11	12
	MISCAN	15,748	15,840	92	15,824	76	16	17
C70 | C3m	CRCSPIN	15,683	15,968	285	15,968	285	0	0
	MISCAN	15,590	15,824	234	15,823	234	1	0
C70 | C9m	CRCSPIN	15,683	15,968	285	15,968	285	0	0
	MISCAN	15,590	15,824	234	15,823	233	1	0
C70 | C18m	CRCSPIN	15,684	15,969	285	15,968	285	0	0
	MISCAN	15,589	15,824	234	15,822	233	2	1
C70 | C@75	CRCSPIN	15,683	15,968	285	15,968	285	0	0
	MISCAN	15,590	15,824	234	15,819	229	5	2
C70 | F3m	CRCSPIN	15,683	15,968	285	15,964	281	5	2
	MISCAN	15,590	15,824	234	15,818	228	6	3
C70 | F9m	CRCSPIN	15,683	15,968	285	15,964	281	4	1
	MISCAN	15,590	15,824	234	15,817	228	7	3
C70 | F18m	CRCSPIN	15,683	15,968	285	15,964	281	4	2
	MISCAN	15,589	15,824	234	15,817	227	7	3
C70 | U	CRCSPIN	15,683	15,968	285	15,930	247	38	13
	MISCAN	15,581	15,815	234	15,726	146	89	38
F70 | F3m	CRCSPIN	15,764	16,024	259	16,023	259	0	0
	MISCAN	15,676	15,902	226	15,902	226	0	0
F70 | F9m	CRCSPIN	15,765	16,024	259	16,023	259	1	0
	MISCAN	15,677	15,903	226	15,903	225	1	0
F70 | F18m	CRCSPIN	15,766	16,025	259	16,024	258	1	0
	MISCAN	15,677	15,903	226	15,903	226	0	0
F70 | U	CRCSPIN	15,764	16,024	259	15,990	226	33	13
	MISCAN	15,677	15,903	226	15,873	196	30	13

Notes: Outcomes calculated over the lifetime of a cohort of 1000 average-risk, CRC-free individuals with age at pandemic defined in the scenario description. The scenario column describes colorectal cancer screening disruption scenarios, as presented in Table 1. Life-years (LY) and Life-years gained (LYG) are computed over the remaining lifespan of individuals starting at the beginning of 2020. All values refer to cohort-level estimates – that is, the expected LY of an average-risk person. This table presents results assuming high colonoscopy sensitivity. Supplementary file 1 presents additional results for the low colonoscopy sensitivity scenario, and Supplementary files 2 and 3 present CRC cases and deaths outcomes.

Similarly, 60-year-olds are expected to incur a substantial reduction in the benefit of screening if screening is discontinued after the pandemic. Those who started screening at age 50 and stopped after the pandemic are expected to lose 106–124 or 92–111 LY per 1000 individuals if pursuing a colonoscopy (C60 | U) or a FIT (F60 | U) screening regimen, respectively. Those who were semi-adherent to FIT screening before the pandemic and discontinued screening (f60 | U) lose even more LY – from 143 to 149 LY per 1000 individuals, or 58–69% of the benefit of screening. Similarly, unscreened 60-year-olds who start screening at age 65 (scenario U60 | C@65) are predicted to lose 42–45 LY per 1000 individuals compared to a scenario where they would have begun screening at age 60 – a 20–22% loss in LYG from screening due to this disruption.

Switching the screening regimen from colonoscopy to FIT and short-term delays will cause only a modest reduction in the benefit of screening. For the 60-year-old cohort, switching from colonoscopy to annual FIT after the pandemic with an 18-month delay is expected to result in a loss of 9–11 LY per 1000 individuals, a 3–4% loss relative to a scenario with no change in screening regimen and no delays. Similarly, short-term delays are predicted to cause minimal decreases in the benefits of the screening program. A 3-month delay in colonoscopy screening results in a loss of 0–2 LY per 1000 individuals for the 60-year cohort (scenarios C60 | C3m), whereas a 9- or 18-month delay (C60 | C9m and C60 | C18m) is expected to result in a loss of 0–2 or 0–3 LY per 1000 individuals, respectively. The worst-case scenario of an 18-month pause starting in March 2020 (scenario C60 | C18m) resulted in a 0–1% loss of the benefit of screening.

Seventy-year-olds lose fewer LY due to screening disruptions but can still be affected by the pandemic as they are at greater risk for CRC than younger age groups. When discontinuing screening after the pandemic, 70-year-olds are expected to lose 38–87 or 29–33 LY per 1000 individuals due to the pandemic if pursuing a colonoscopy (C70 | U) or FIT (F70 | U) screening regimen, respectively. Unscreened 70-year-olds who only come back to screening at age 75 (scenario U70 | C@75) are expected to lose 49–50 LY per 1000 individuals, a 39–43% reduction in LYG relative to a scenario where they would have received colonoscopy screening at age 70.

Seventy-year-olds who were up-to-date with their screening and experienced short-term delays of up to 18 months can expect minimal loss of LY due to pandemic-induced CRC screening disruptions, even if they switch to FIT after the pandemic. Those who transitioned from colonoscopy to FIT screening at age 70 can expect a reduction of 5–7 LY per 1000 individuals even if a return to FIT screening was delayed by 18 months (scenario C70 | F18m). This reduction in benefit represents a 2–3% reduction in LYG of colonoscopy-only screening.

### Low-sensitivity scenarios

**Figure 2.**
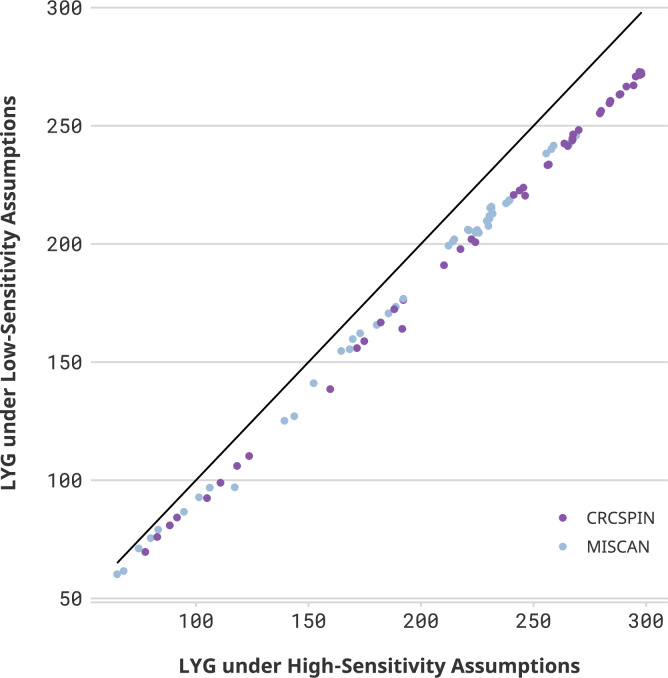
Life-years gained (LYG) in high- vs. low-sensitivity scenarios. Notes*:* Each dot represents one scenario considered in this study. The horizontal axis displays the number of LYG estimated in that scenario under a high colonoscopy sensitivity scenario. The vertical axis shows the results for the same cohort under a low colonoscopy sensitivity scenario. If sensitivity did not affect the estimate, then all points would be on top of a 45-degree line. Different colors represent CRCSPIN and MISCAN models.

While colonoscopy sensitivity affects the overall benefit of screening, *conditional on colonoscopy sensitivity*, the loss of LY due to pandemic-induced scenarios is similar across sensitivity levels. Figure 2 compares LYG and LYL for high- and low-sensitivity scenarios. High-sensitivity scenarios are expected to yield higher LYG benefits than low-sensitivity scenarios, and the magnitude of this difference is higher for more intensive screening regimens. For 60-year-olds with a prior colonoscopy at age 50 who experience an 18-month delay during the pandemic (scenario C60 | C 18m), the benefit of screening is 240–297 LYG per 1000 individuals under a *high colonoscopy sensitivity* scenario, whereas it is 217–272 under a *low colonoscopy sensitivity* scenario.

**Figure 2—figure supplement 1.**
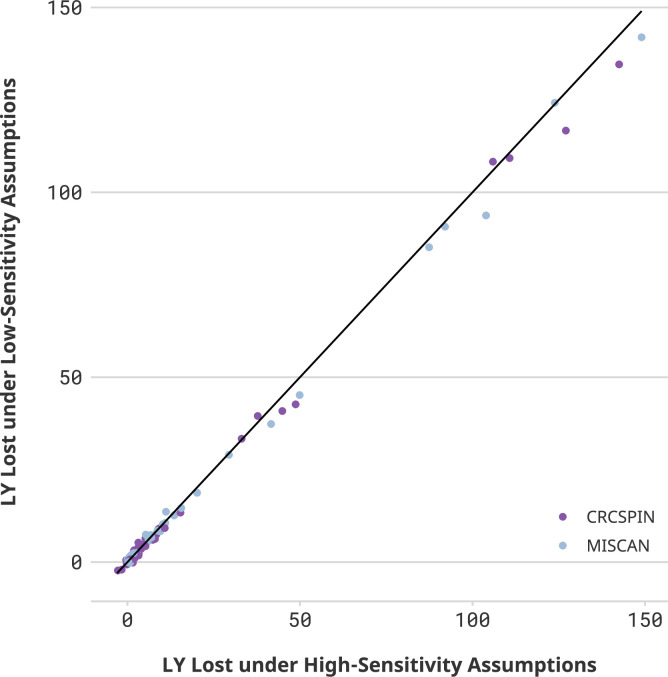
Life-years lost in high- vs. low-sensitivity scenarios. Notes: Each dot represents one scenario considered in this study. The horizontal axis displays the number of life-years lost due to disruptions (LYL) estimated in that scenario under a high colonoscopy sensitivity scenario. The vertical axis shows the results for the same cohort under a low colonoscopy sensitivity scenario. If sensitivity did not affect the estimate, then all points would be on top of a 45-degree line. Different colors represent CRCSPIN and MISCAN models.

Nevertheless, conditional on the sensitivity scenario, the effect of pandemic disruptions on LY lost is expected to be very similar for low-sensitivity scenarios. An 18-month delay in colonoscopy screening is expected to result in a loss of 0–3 LY per 1000 individuals for 60-year-olds assuming high sensitivity, whereas it is expected to result in a loss of 0–4 LY per 1000 individuals assuming low sensitivity.

## Discussion

Model-based screening cost-effectiveness analyses present estimates under guideline-concordant scenarios, but there are many reasons why real-world screening will not follow guidelines. Chief among them in 2020, the COVID-19 pandemic severely disrupted screening. Under those conditions, disparities in health outcomes can arise if disruptions are unevenly distributed in the population.

Our results suggest that the COVID-19 pandemic will have an uneven effect on CRC outcomes depending on whether and how fast screening is resumed after the pandemic onset. Consider three cohorts with the same pre-pandemic screening regimen and behavior: 60-year-olds with a prior colonoscopy at age 50. Cohorts that experience short-term disruptions (e.g. 3–18 months) only experience a small loss of life due to short-term delays – up to 3 LY per 1000 individuals. Those who switch from colonoscopy to FIT screening are projected to experience a greater loss of life – from 9 to 11 to LY per 1000 individuals. If screening is only resumed at age 65 (e.g. age at Medicare enrollment) or abandoned, the loss of benefits from screening could be 3–11 LY per 1000 individuals (scenario C60 | C@65). Lastly, discontinuing screening after the pandemic is projected to cause a loss of 106–124 LY per 1000 individuals, a decrease of 36–51% in the benefit of screening (scenario C60 | U). These results imply that the pandemic will become a disparity-widening mechanism if it differentially affects screening access and/or behavior across different population groups. These results also show that the pandemic is unlikely to substantially affect those whose screening is only interrupted momentarily.

These results highlight the potential implications of disruptions to preventative care due to loss of insurance following the pandemic. According to data from the Bureau of Labor Statistics Current Population Survey, more dramatic declines in the number employed during the COVID-19 pandemic were seen in Black, Asian American, and Hispanic groups (Gemelas et al., 2022). Moreover, data from the US Census Household Pulse Survey suggests that Black and Hispanic workers were not only more likely to be unemployed but were also more likely to be without unemployment insurance (Mar et al., 2022). These results provide important clinical insight on the projected impact of these populations which may guide future policy on the aftereffects of the pandemic. Those who were previously uninsured for long periods of time throughout the pandemic should resume CRC screening to mitigate the long-term effects projected in these simulations.

These results also add to the growing evidence of the implications of delayed CRC care following the COVID-19 pandemic. A microsimulation study based on a Canadian population explored scenarios of differing screening delays and transition periods due to attenuated screening volumes and found that a 6-month delay in primary screening could increase CRC incidence by 2200 cases and 960 more cancer deaths over a lifetime (Yong et al., 2021). A microsimulation paper based on a Chilean population illustrated similar results with respect to CRC incidence and mortality due to the screening backlog and strained patient care during the pandemic (Ward et al., 2021). Our results mirror these conclusions and provide new scenarios which consider the aftereffects of loss of healthcare insurance due to disparities magnified by the COVID-19 pandemic.

### Limitations

This analysis presents a series of limitations. First, we do not present population-level estimates of reductions in benefits. While doing so could prove helpful, one would have to estimate how many people will be screened following each scenario we modeled. That would require individual-level data describing the distribution of delays and screening regimen switching in the population after the pandemic, which will not be available for many years. Instead of pursuing a population-level study, we conditioned our estimates on a discrete set of pre-specified disruption scenarios. This approach makes our study feasible but prevents us from making population-level predictions. Moreover, our approach does not account for potential correlation between risk factors and disruptions – we only provide estimates using models calibrated to represent cohorts with average risk.

Second, the scenarios presented in this analysis represent only a subset of the real-world changes in screening due to the pandemic. Even in the absence of a pandemic, individuals may switch from colonoscopy to FIT, and return to colonoscopy screening. To keep this analysis tractable, we restrict the variations considered in this paper to one switch from colonoscopy to FIT. Further, we only consider changes in screening regimens immediately following the COVID-19 pandemic. Third, this analysis only considers uncertainty stemming from structural differences between models and two scenarios of test characteristics and does not evaluate parameter or sampling uncertainty. Our estimates represent the expected value of estimates conditional on scenarios across an average-risk cohort drawn from the general US population.

Finally, this paper identifies the effect of disruptions on the effectiveness of screening interventions but does not explicitly identify policy interventions or prioritization rules to amend those inequities. Future research could use extended cost-effectiveness analysis to evaluate CRC screening interventions in the context of healthcare disparities (Asaria et al., 2016; Richard et al., 2020).

### Conclusion

This study quantified the potential effect of disruptions to colonoscopy screening and demonstrated that unequal recovery of CRC screening following the pandemic will predictably widen disparities in CRC outcomes. The COVID-19 pandemic will severely reduce the benefits of CRC screening if it causes screening discontinuation or long-term (e.g. 5 year) delays. Short-term delays of 3–18 months and regime switching from colonoscopy to FIT are not expected to have significant consequences.

## Data Availability

This is a computational study based on two independently developed simulation models. Simulation output data and code used to produce the figures and Supplementary Table 3 in this paper are available at https://github.com/c-rutter/unequal-recovery-covid-19, (copy archived at swh:1:rev:0244d3219552031e056f7b5d6aa02fe03276080d). Full documentation of CISNET models used to produce the results presented in this study can be found at https://cisnet.cancer.gov/colorectal/profiles.html. Interested researchers can contact authors directly for more insight into the CISNET models.

## Additional files

MDAR checklist

Supplementary file 1. Projected lifetime life-years (LY) per 1,000 individuals.

Supplementary file 2. Projected lifetime CRC cases per 1,000 individuals.

Supplementary file 3. Projected lifetime CRC deaths per 1,000 individuals.
